# Co-infection with *Schistosoma mansoni* and Human Immunodeficiency Virus-1 (HIV-1) among residents of fishing villages of north-western Tanzania

**DOI:** 10.1186/s13071-014-0587-2

**Published:** 2014-12-16

**Authors:** Humphrey D Mazigo, David W Dunne, Shona Wilson, Safari M Kinung’hi, Angela Pinot de Moira, Frances M Jones, Domenica Morona, Fred Nuwaha

**Affiliations:** Department of Medical Parasitology and Entomology, Weill School of Medicine, Catholic University of Health and Allied Sciences, P.O. Box 1464, Mwanza, Tanzania; Department of Disease Control and Environmental Health, School of Public Health, College of Health Sciences, Makerere University, P.O. Box 7072, Kampala, Uganda; Department of Pathology, Division of Microbiology & Parasitology, Cambridge University, Tennis Court Road, Cambridge, CB2 1Q3 P UK; National Institute for Medical Research, Mwanza Research Centre, P.O. Box 1462, Mwanza, Tanzania

**Keywords:** *S. mansoni*, HIV-1, Co-infection, Fishing villages, Risk factors, Tanzania

## Abstract

**Background:**

Co-infection with *S. mansoni* and Human Immunodeficiency Virus-1 (HIV-1) has been described in sub-Saharan Africa. However, few community-based studies have been conducted to assess the association between the two diseases. The present study examined whether the infection with HIV-1 is associated with an altered susceptibility to *S. mansoni* infection by comparing the prevalence and intensity of *S. mansoni* infection among those infected and not infected with HIV-1. Any influence of HIV-1 associated immunodeficiency on the intensity of *S. mansoni* infection was also investigated.

**Methods:**

A cross-sectional study was conducted among 1,785 randomly selected adults (aged 21–55 years) in fishing villages of north-western Tanzania. Single stool samples were obtained and examined for *S. mansoni* eggs using the Kato Katz technique. Finger prick and venous blood samples were collected for HIV-1 screening and CD4^+^ cell quantification. Demographic information was collected by questionnaire.

**Results:**

Of the 1,785 individuals from whom complete data were obtained, 854 (47.85%, 95% CI; 40.46 – 56.57) were infected with *S. mansoni* and had a mean intensity of 183.21(95% CI; 165.61-202.70) eggs per gram of faeces (epg). A total of 125 individuals (6.29%, 95% CI 3.59-11.04) were infected with HIV-1 and only 40% (n=50) of them were co-infected with *S. mansoni.* No differences in prevalence of *S. mansoni* infection or intensities of infection, as estimated by egg count (epg), were observed between HIV-1 sero-positive individuals and HIV-1 negative individuals. In generalized regression models (adjusted for sex, age, occupation, residence and level of education), being infected with HIV-1 did not increase the risk (APR=1.01, 95%; 0.83-1.21, *P*=0.93) or intensity (AOR = 0.84, 95% CI; 0.56-1.25, *P =* 0.33) of *S. mansoni* infection. Among individuals co-infected with HIV-1 and *S. mansoni* infection, the intensity of infection (epg) was not associated (*P =* 0.21) or correlated (*P =* 0.13) with CD4^+^ cell counts.

**Conclusion:**

Our findings suggest that HIV-1 infection may not have a major effect on *S. mansoni* infection or on the excretion of eggs from the co-infected individuals. However, further studies are needed to understand the biological interaction between HIV-1 and *S. mansoni* in a large cohort of co-infected individuals.

## Background

In the past two decades, epidemiological studies have reported an overlap of Human Immunodeficiency (HIV-1) and *S. mansoni* infections in sub-Saharan Africa [1-3], leading to co-infection in highly endemic regions [1-4]. *Schistosoma mansoni* is focally distributed in the region, its distribution being largely influenced by the distribution of its intermediate snail host and human–water contact behaviours [5,6]. In addition, the development of its related morbidities is influenced by genetic factors of the affected population [7], immune responses against the parasites or eggs [8] and changes in body physiology with increased age [9]. These factors may influence the prevalence and intensity of infection patterns in endemic populations. In contrast, HIV-1 is widely distributed and its transmission involves a combination of multiple factors including demographic factors and socio-economic status, epidemiological settings (rural *versus* urban) and sexual behaviours [10-12].

In areas where *S. mansoni* is highly endemic, such as in fishing villages along the Lake Victoria shores in east Africa [13], the migratory behaviour of fishermen and women [14], multiple sexual partner networks [11], living in clusters and isolated localities away from basic health services, a high prevalence of sexual transmitted diseases [15] and social cultural behaviours [11,12,15] increase the risk of HIV-1 transmission. The overlap of multiple risk factors associated with the two diseases in the same geographical setting or the biological interaction between HIV-1 and *S. mansoni* [2,10-12,14] have been proposed to increase the risk of individuals to be co-infected with both HIV-1 and *S. mansoni* [2,11,13,15]. In co-infected individuals, immunological studies have described a number of biological mechanisms through which chronic HIV-1 infection could affect *S. mansoni* related morbidities [16]. These mechanisms could result in differences in the prevalence and intensity of *S. mansoni* infection [17,18]; the efficiency of parasite egg excretion [4,17,18]; morbidity patterns [19] and the response to anthelmintic treatment among HIV-1 infected and non infected people [20,21]. However, despite the possible interaction between the two diseases, few community based studies have been conducted to explore the possible interaction between HIV-1 and *S.mansoni* infections [4,17,18]. The current study examined whether infection with HIV-1 is associated with an altered susceptibility to *S. mansoni* infection by comparing the prevalence and intensity of *S. mansoni* infection among those infected and not infected with HIV-1. As HIV-1 infection is associated with reduced CD4^+^ cell counts, and as the human immunological status is known to influence schistosome-human reactions, we also tested if there was an association or correlation between the intensity of *S.mansoni* infection and the immunological status, as measured by CD4^+^ cell counts, in people co-infected with HIV-1 and *S. mansoni*.

## Methods

### Study population and study area

The study was conducted at Ilemela district, Mwanza region, located at 32-34°E and 2 - 4°S, on the southern shores of the Lake Victoria, north-western Tanzania. The area experiences a temperature range from 18°C to 28°C and the mean annual rainfall of 1068 mm. Four villages Sangabuye, Kayenze, Igalagala and Igombe, were chosen for the present study due to their close proximity to the lake. The majority of the population are of Sukuma tribe. Other migrant tribes are Kerewe, Jita, and Kara. The majority of the villagers in the four villages depend on the lake for domestic and economic activities such as washing, bathing, cooking, drinking and recreation. The main economic activities in the four villages are fishing and farming. For high water contact levels, the residents have a high risk of being infected with *S. mansoni* [22,23] and high occupational exposure maintains high intensities of *S. mansoni* infection into adulthood [24]. Annual mass drug administration (MDA) using praziquantel and albendazole against helminth infections in these villages is organised within the school environment and focuses on school-going children, not the adult population.

The high rate of sexual mixing within the fishing villages increases the risk of HIV-1 transmission in the adult population [11]. In 2003, the prevalence of HIV-1 infection in individuals aged 15–60 years living in Kayenze and Sangabuye villages was estimated to be 10% [22] and the National HIV-1 seroprevalence results of 2012 have indicated that the prevalence of HIV-1 is 6% in the age group 15–60 years [25].

### Study design, inclusion and exclusion criteria

This cross-sectional study was conducted between September 2012 and December 2012. All people who had lived in the study villages for more than two years, and were aged 21 to 55 years, were eligible for enrolment. Individuals with a history of treatment for schistosomiasis (praziquantel) in the past six months and those who were on anti-retroviral treatment were excluded from the study.

To determine an adequate sample size, we assumed that among people not infected with HIV-1, 50% were infected with *S. mansoni*. In order to determine a difference of 20% among people with HIV-1 who were infected with *S. mansoni,* at a power of 80% and 95% confidence interval, we required at least 103 people infected with HIV-1. This sample size was calculated using Stata 12 using the command sampsi 0.5 0.7, power (.80). To achieve this number of HIV infected we needed to screen 1,760 people assuming an HIV-1 prevalence of 6% [25] and refusal rate of 10%.

A two-step sampling procedure was used to select households and household members to participate in the study. At first, random sampling was used to select 1,785 households from a list of 4586 households. Then, from every selected household, random sampling was used to select one individual from all eligible persons in the household. If the selected individual was not present on the day of screening, the house was revisited three times on different days and if the selected subject declined to participate or was not available after multiple attempts, a new member of the household was chosen. Similarly, if the selected household remained vacant after multiple visits, the household was substituted by a neighbouring one.

### Data collection

(i) **Human Immunodeficiency Virus screening and CD4**^**+**^**analysis**

Human Immunodeficiency Virus-1 testing was conducted according to the Tanzanian National HIV algorithms which recommend the use of a rapid test qualitative immunoassay [26]. Study participants were counselled before and after HIV testing as per requirement of the country algorithms. The study used Determine® (Alere Determine, Chiba, Japan) followed by UNI-GOLD® (Trinity Biotech PLC, Bray, Ireland) for the participants who had a positive result with Determine [26]. The test was conducted according to the standards provided by the manufacturers. For individuals who had a Determine positive test but a negative UNIGOLD test, a dry blood spot was obtained for Enzyme Immunosobent Assay (ELISA) testing. Within 24-hours of blood sample collection, the quantification of CD4^+^ cells was carried out using a FACSCalibur machine (Becton Dickinson-BD Biosciences, San Jose, CA, USA) following standard procedures [27].

(ii) **Parasitological screening for*Schistosoma mansoni***

A single stool sample was collected from all study participants. Four Kato Katz thick smears were prepared from different parts of the single stool sample using a template of 41.7 mg (Vestergaard Frandsen, Lausanne, Switzerland), following a standard protocol [28-30]. After 24 hours, the smears were independently examined for *S. mansoni* eggs by two experienced laboratory technicians of the National Institute for Medical Research (NIMR) laboratory. For quality assurance, a random sample of 10% of the negative and positive Kato Katz thick smears were re-examined by a third technician.

In addition to stool and blood examination, demographic information on sex, age, occupation, marital status, village of residence, number of years lived in current residence and level of education were collected by questionnaire.

### Ethical considerations

Ethical approval was obtained from the Higher Degrees Research and Ethics Committee of the School of Public Health, Makerere University (Institutional Review Board (IRB) -00005856/2011) and from the Bugando University College of Health Sciences and Allied Sciences-Institutional Review Board, (BREC/001/32/2011). Ethical clearance was granted by the National Ethical Review Committee, National Institute for Medical Research, Tanzania and the study was registered in the clinical trial network, Clinical Trial (Number:- NCT-01541631). The study received further clearance from the regional and district administrative authorities of Mwanza region and Ilemela district. Swahili translated informed assent and consent forms were used to obtain children and adult participants’ consent respectively. For illiterate individuals, a thumb print was used to sign the assent and consent forms after a clear description of the study objective and acceptance to participate.

All study participants who were infected with *S. mansoni* were treated with praziquantel (40 mg/kg) according to World Health Organization (WHO) guidelines, irrespective of their HIV-1 sero-status. All HIV-1 infected individuals found to have CD4^+^ < 350 cells/μL were referred to the Care and Treatment Clinic (CTC) for assessment of their eligibility for antiretroviral therapy (ART).

### Statistical analysis

The data were double entered using CSPro and the final data set was stored in a MySQL database. Data analysis was performed using Stata version 12 (Stata Corp, College station, Texas, USA). The prevalence of *S. mansoni* 95% confidence interval (95% CI) were obtained by binomial logistic regression taking into account clustering by villages. Comparisons of prevalence by demographic factors for *S. mansoni* infection were tested for significance using the chi-square test (χ^2^) or the Fisher exact test for categorical variables before running regression models. The arithmetic mean of *S. mansoni* egg counts for each participant was calculated from the counts of four Kato Katz thick smears and multiplied by 24 to obtain individual eggs per gram of faeces. *S. mansoni* egg counts were over dispersed so were logarithmically transformed and used to calculate the geometric mean egg per gram of feaces (GM-epg) that was obtained as the antilog of the mean of the transformed egg counts. Geometric mean egg counts for *S. mansoni* between various demographic factors were compared using t-test or ANOVA. Intensity of infection was categorized according to WHO criteria as : 1–99 epg, 100–399 epg, ≥400 epg defined as low, moderate and heavy intensities of infection respectively [29,30]. To test for association between intensity of *S. mansoni* infection and degree of immunosupression with HIV-1 as measured by CD4^+^ cell counts, linear regression analyses were done to calculate Pearson correlation coefficient for individuals co-infected with *S. mansoni* and HIV-1 adjusting for sex and age.

We also assessed whether using cut-offs of 350 and 200 CD4^+^ cells/μL, which are used for treatment referral and as a definition of Acquired Immunodeficiency Syndromes (AIDS) [31] respectively, influence susceptibility with *S.mansoni*. To control for the confounding effects of age, sex, occupation, marital status, level of education, village of residences and duration of residence on the association between HIV-1 and *S. mansoni* infections binomial logistic and generalized regressions models were done for all people whether infected with *S. mansoni* or not. Stepwise backward procedures were used to determine whether these variables were independent factors of *S. mansoni* infection by use of adjusted prevalence ratio (APR) for logistic regression. To control for confounding regarding intensity of infection, linear regression models were run for people who were infected with *S .mansoni*. Stepwise backward procedures were used to determine whether these variables were independent factors of intensity of *S. mansoni* infection by using adjusted odds ratios (AOR) for linear models and the 95% confidence interval (CI).

## Results

(i) **Demographic characteristics of study participants round decimals to one point**

A total of 1,785 individuals who participated in this study, 52.9% (945/1785) were female and 47.0% (n = 840) were male. The mean age of the study participants was 35.6 ± 9.74 years (with no significant difference in mean age between males and females, *P =* 0.4). The majority of the study participants had received a primary school education n = 1078, (60.39%), n = 76 (4.3%) had attained secondary/college education, n = 631, (35.4%) were illiterate. The majority were married (n = 1,257 (70.9%), 234 (13.1%) reported being in a long-term, stable relationship and 291 (16.4%) were either separated or divorced/widowed. There were 1,256 (70.36%) farmers, 255 (14.3%) fishermen and 223 (15.4%) small-scale business men and women. Overall, 97% of the study participants performed their economic activities within their villages.

(ii) **Prevalence of*Schistosoma mansoni*infection and association with HIV-1**

The overall prevalence of *S. mansoni* was 854/1,785 (47.84%, 95%CI, 40.46-56.57), which was higher among males than females (472/840, 56.19% *versus* 382/941, 40.59%, *P <* 0.0001) (Table 1). There was a relationship between age and *S. mansoni* infection, the older age had lower prevalence compared to young age groups (*P <* 0.00001). Fishing-related occupations were associated with a higher prevalence of infection compared to other economic activities (*P <* 0.0001). Similarly, the prevalence varied significantly by villages of residence (*P <* 0.0001), with inhabitants of Kayenze and Igalagala having the highest prevalence. However, *S. mansoni* infection did not vary by duration of residence (Table 1). In relation to HIV-1 serostatus, 50/125 (7.00%) were co-infected with HIV-1 and *S. mansoni* infection. HIV-1 seronegative individuals had a higher prevalence of *S. mansoni* infection compared to the HIV-1 seropositive individuals but this difference was not statistically significant (*P =* 0.06) (Table 1). In generalised regression models (Table 1) adjusted for sex, age, marital status, education levels, occupation, village of residence, and duration of stay, HIV-1 was not an independent risk factor for infection with *S. mansoni* (APR = 1.01,95% CI; 0.84 - 1.21, *P =* 0.93). The main independent risk factors of *S. mansoni* infection were being of the male gender (APR = 1.27, 95% CI;1.14-1.42, *P <* 0.001), involvement in fishing activities (APR = 1.28,95% CI;1.07–1.53, *P <* 0.01), living in Igalagala (APR = 1.31,95% CI;1.12-1.52, *P <* 0.001) and Kayenze (APR = 1.32,95% CI;1.15-1.51, *P <* 0.001) villages.

**Table 1:** **Prevalence and risk factors of** ***Schistosoma mansoni*** **among residence of fishing villages of north-western Tanzania**

**Variable**	**No. infected**	**Prevalence of** ***S. mansoni*** **(95% CI)**	**Crude prevalence ratio (95% CI)**	**Adjusted prevalence ratio (95% CI)**
**Overall**	854	47.84 (40.41 – 56.60)	-----	-----
**Sex**				
Female	382	40.29 (32.43 – 50.00)	1	1
Male	472	56.14 (49.19 – 64.06)*	1.39 (1.26 – 1.54)**	1.27 (1.14 – 1.42)***
**Age (in years)**				
21 – 30	365	53.18 (47.48 – 59.55)*	1.22 (1.99 – 1.48)	1.19 (0.98 – 1.45)
31 – 40	292	47.66 (38.38 – 59.19)	1.09 (0.99 – 1.19)	1.12 (0.92 – 1.37)
41 – 50	125	38.96 (29.80 – 50.93)	0.89 (0.76 – 1.04)	0.91 (0.72 – 1.13)
51 – 55	72	43.71 (35.49 – 53.83)	1	1
**Marital status**				
Married	591	47.20 (36.04 – 61.83)	1	1
Single	263	48.63 (29.60 – 79.87)	1.03 (0.82 – 1.29)	1.05 (0.95 – 1.16)
**Education level**				
Literate	564	49.04 (43.27 – 55.59)	1	1
Illiterate	290	45.44 (34.83 – 59.28)	0.93 (0.91 – 0.94)	0.98 (0.88 – 1.08)
**Occupation**				
SME	129	44.14 (36.14 – 53.91)	1	1
Peasants	561	45.11 (37.98 – 53.58	1.02 (0.89 – 1.16)	1.06 (0.86 – 1.24)
Fishing	164	66.28 (55.77 – 78.78)*	1.52 (1.42 – 1.59)**	1.27 (1.06 – 1.53)***
**Village of residences**				
Igombe	176	40.42 (29.82 – 54.78)	1	1
Igalagala	141	55.47 (39.08 – 78.72)*	1.37 (1.31 – 1.44)**	1.31 (1.12 – 1.52)***
Kayenze	351	54.21 (42.96 – 68.38)*	1.34 (1.25 – 1.44)**	1.32 (1.15 – 1.51)***
Sangabuye	186	41.24 (26.34 – 64.58)	1.02 (0.88 – 1.78)	1.06 (0.89 – 1.25)
**Duration of residences (in years)**				
3 – 5	152	50.16 (43.42 – 57.94)	1	1
6 – 10	169	50.21 (38.90 – 64.79)	1.01 (0.80 – 1.25)	1.01 (0.86 – 1.18)
11 – 20	187	49.76 (38.92 – 63.61)	0.98 (0.85 – 1.16)	1.01 (0.88 – 1.16)
≥21	346	48.69 (43.06 – 55.06)	0.97 (0.92 – 0.99)	0.96 (0.85 – 1.09)
**CD4** ^**+**^ **cell counts/μL**				
<350	28	38.36 (28.63 – 51.38)	1	-----
≥350	22	44.89 (28.96 – 69.61)	1.17 (0.91 – 1.51)	-----
**HIV-1 sero-status**				
Negative	804	48.12 (41.04 – 56.43)	1	1
Positive	50	39.52 (27.64 – 56.49)	0.92 (0.78 – 1.09)	1.01 (0.84 – 1.21)

*P < 0.001 from χ^2^; **P < 0.001 and ***P < 0.001 from binomial family model.

(iii) **Intensity of*S.mansoni*infection and its association with HIV-1**

The overall geometrical mean egg per gram of faeces (GM-epg) for study participants with detectable *S. mansoni* eggs was 182.85epg (95% CI: 165.27 – 202.31). Males had higher intensities of infection (238.31epg, 95% CI: 206.63 – 274.84) than females (132.07epg, 95% CI: 115.29 – 151.28, *P <* 0.0001) (Table 2). The youngest age group (21–30 years) had the highest intensities of infection than older age groups (Table 2). Also, those involved in fishing activities had the highest intensities of infection compared to other economic activities (*P <* 0.0012). In addition, illiterate individuals had the highest intensity of infection compared to literate individuals (*P <* 0.034). Inhabitants living in Kayenze and Igalagala villages had the highest intensities of infection compared to those who were living in the other two villages (*P <* 0.002) (Table 2). Of the 854 individuals with detectable *S. mansoni* eggs, 352/854 (41.21%), 232/854 (27.16%) and 270/854 (31.61%) had light, moderate and heavy infections respectively, as defined by WHO [29,30].

**Table 2:** **Factors associated with infection intensity among people infected with** ***S.mansoni*** **residing in fishing villages of north-western Tanzania**

**Variable**	**No. infected**	**GM-epg (95% CI)**	***F*** **-ratio (** ***P*** **-level)**	**COR (95% CI)**	**AOR (95% CI)**
Overall	854	183.21 (165.61 – 202.70)	------	-------	-------
**Sex**					
Female	382	132.02 (115.33 – 151.14)	− 4.7386* (*P <* 0.0001)	1	1
Male	472	238.86 (207.14 – 275.44)		1.61 (1.48 – 2.21)	1.65 (1.32 – 2.08)
**Age (in years)**					
21 – 30	368	220.20 (188.16 – 257.71)	2.03** (*P =* 0.11)	1.61 (0.65 – 1.54)	1.68 (1.14 – 2.48)
31 – 40	286	177.55 (149.10 – 211.41)		1.30 (0.89 – 1.91)	1.32 (0.89 – 1.97)
41 – 50	127	136.85 (109.18 – 171.54)		1.004 (0.65 – 1.91)	1.08 (0.69 – 1.67)
51 – 55	73	136.85 (109.18 – 171.54)		1	1
**Marital status**					
Married	591	173.06 (153.51 – 195.09)	− 0.8094* (*P =* 0.42)	1	1
Single*	263	204.46 (168.53 – 248.04)		1.02 (0.93 – 1.04	0.99 (0.94 – 1.05)
**Occupation**					
SSB	129	141.93 (108.59 – 185.21)	6.75** *P <* 0.0012	1	1
Peasants	561	161.72 (142.87 – 183.06)		1.14 (0.82 – 1.57)	1.12 (0.81 – 1.54)
Fishing	164	308.84 (246.20 – 387.41		2.18 1.50 – 3.15)	1.62 (1.10 – 2.41)
**Education level**					
Literate	564	173.54 (153.35 – 196.38)	−2.1185* (*P <* 0.0344)	1	1
Illiterate	290	197.38 (164.80 – 236.40)		1.14 (0.91 – 1.41)	1.33 (1.07 – 1.66)
**Village of residence**					
Igombe	176	144.14 (116.73 – 178.00)	3.26** (*P <* 0.0211)	1	1
Igalagala	141	224.83 (175.65 – 287.77)		1.56 (1.12 – 2.17)	1.71 (1.23 – 2.40)
Kayenze	351	202.39 (173.12 – 236.61)		1.40 (1.07 – 1.84)	1.62 (1.23 – 2.13)
Sangabuye	186	163.07 (129.35 – 205.58)		1.13 (0.83 – 1.54)	1.38 (1.001 – 1.89)
**Duration of residence (in years)**					
3 − 5	152	230.94 (180.85 – 294.91)	1.89** (*P =* 0.13)	1	1
6 – 10	169	160.53 (122.74 – 209.95)		0.69 0.48 – 1.01	0.71 (0.50 – 1.01)
11 – 20	187	188.01 (154.38 – 228.95)		0.81 (0.59 – 1.11)	0.86 (0.64 – 1.17)
≥21	346	165.24 (141.06 – 193.58)		0.71 (0.54 – 0.95)	0.73 (0.55 – 1.04)
**CD4** ^**+**^ **cell counts/μL**					
<350	28	154.27 (85.16 – 279.48)	1.5410* (*P =* 0.13)	1	
≥350	22	93.26 (60.42 – 143.95)		1.09 (0.54 – 2.08)	------
**HIV-1 serostatus**					
Negative	804	186.83 (168.15 – 207.59)	0.882* (*P =* 0.38)	1	1
Positive	50	126.78 (86.59 – 185.61)		0.76 (0.51 – 1.13)	0.84 (0.56 – 1.25)

● Determined by based on t-tests **Determined by Anova.

● COR = Crude Odd Ratio.

● AOR = Adjusted Odd Ratio.

● Single* - male of female individuals living alone/not married.

HIV-1 seronegative individuals had higher intensities of infection (186.98epg, 95% CI; 168.39-207.61) than HIV-1 seropositive individuals (126.78epg, 95% CI; 86.59 – 185.61) but the observed difference did not reach significance (*P =* 0.38). In a multiple linear regression model adjusted for age, sex, occupation, marital status, education level, residence and duration of stay, the intensity of *S. mansoni* infection was not associated with HIV-1 infection (AOR = 0.84,95% CI; 0.56 – 1.25) (Table 2). The intensity of infection was associated with being of male gender (AOR = 1.65,95% CI; 1.32-2.08, *P <* 0.0001), young ages (21–30 years; AOR = 1.68,95% CI; 1.14-2.48, *P <* 0.01), involvement in fishing activities (AOR = 1.62,95% CI; 1.10-2.41, *P <* 0.01), being illiterate (AOR = 1.33,95% CI; 1.07 – 1.66, *P <* 0.014) and living in the study villages.

(iv) **Association between immune status and*S. mansoni*infection**

Of the 125 individuals infected with HIV-1, 50/125, (40.00%, 95% CI; 31.58 - 48.42) had detectable *S. mansoni* eggs in their stool samples. The overall prevalence of co-infection for the entire cohort was 50/1,785 (2.80%). Of the co-infected individuals, the proportions of lightly, moderately and heavily co-infected individuals were 25/50, (50.00%, 14/50 (28.00%) and 11/50 (22.00%) as defined by WHO [29,30]. The prevalence of *S. mansoni* in HIV-1 infected individuals with CD4^+^ counts <350 cells/μL was 28/75 (37.33%) compared to 22/50 (44.00%) in those who had CD4^+^ counts ≥350 cells/μL (*P =* 0.47). Co-infected individuals with CD4^+^ counts <350 cells/μl had a higher GM-epg of *S. mansoni* infection (154.27epg, 95% CI; 85.16-279.48) compared to those who had CD4^+^ counts ≥350 cells/μl (93.26 GM-epg, 95% CI: 60.42- 143.95), but the difference was not significant (*P =* 0.13). Among those with CD4^+^ counts <200 cells/μL, 24.44% (n = 11) were co-infected with HIV-1 and *S. mansoni,* while among those with CD4^+^ cell counts ≥ 200 cells/μL, 50.65% (n = 39) were co-infected. Considering the intensities of *S. mansoni* infection, those who had CD4^+^ counts of <200 cells/μL had higher GM-epg of *S. mansoni* infection (184.83epg, 95% CI: 54.85-622.82), compared to those who had CD4^+^ counts of ≥200 cells/μL (110.376epg, 95% CI; 75.63 – 161.07), but the difference between the two groups did not reach significance (*P =* 0.13).

For individuals who were mono-infected with HIV-1, 45/75 (60.00%) had CD4^+^ counts <350 cells/μL and 30/75 (40.00%) had CD4^+^ counts ≥ 350 cells/μL. The median CD4^+^ counts for individuals who were infected with HIV-1 only was 231.5(range: 132–512.5) cells/μL and those who were co-infected with *S. mansoni* and HIV-1 was 308.5 (range: 202–586) cells/μL (*P =* 0.14).

**Figure 1:**
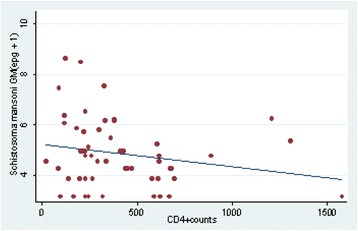
**Scatter plot of correlation of** ***S. mansoni*** **egg counts and CD4** ^**+**^ **counts in individuals co-infected with HIV-1 and** ***S. mansoni*** **.** No correlation was observed between egg excretions and CD4^+^ counts levels (r = −0.37, *P =* 0.13).

For individuals who were co-infected, the intensity of infection was not associated with CD4^+^ count at both univariate (*P =* 0.11) and multivariate level (*P =* 0.21) (correcting for age, sex, village of occupation, residence, education level) (R^2^ = 0.3027, adjusted R^2^ = 0.1458, F = 0.075). No correlation was observed between intensity of *S. mansoni* infection (epg) and CD4^+^ cell counts among the co-infected individuals (r = −0.37, *P =* 0.13) (Figure 1).

## Discussion

The present study villages of North-western Tanzania are endemic to *S. mansoni* and HIV-1 infections. Co-infection with HIV-1 and *S. mansoni* in a single human host does also occur in the study population. In the present study area, no differences in prevalence of *S. mansoni* infection or egg counts were observed between HIV-1 sero-positive participants and HIV-1 sero-negative individuals. After adjusting for other factors in the statistical models, no association was observed between the HIV-1 serostatus and the prevalence as well as the intensity of *S.mansoni* infection*.* Furthermore, no correlation was observed between CD4^+^ cell counts and *S. mansoni* egg counts.

Our observations were consistent with the study of Kallestrup *et al.* [18], in rural Zimbabwe; although that study was limited by low intensities of *S. mansoni* infection, no difference in egg counts or prevalence was observed between HIV-1 seropositive or HIV-1 sero-negative participants infected with *S. mansoni* [18]. These observations are inconsistent with reports from previous similar studies in Ethiopia and Western Kenya [4,17], where individuals co-infected with HIV-1 and *S. mansoni* infections excreted fewer egg counts per gram of faeces than HIV-1 negative individuals infected with *S. mansoni* despite the two groups having similar worm burdens [4], as determined by Circulating Cathodic Antigen (CCA) levels (a *S. mansoni* gut antigen which is regurgitated by juvenile and adult stages as a by-product of host red blood cell digestion) [32]. Complementary to these observations in human hosts, a lower egg output in immunodeficient murine models has also been described [33-38]. In these models, the maturation of the worm is delayed; the fecundity of the female worm affected and transposition of the eggs to the lumen of the intestine is reduced. These combine to significantly lower the level of *S.mansoni* egg output compared with immunocompetent mice, despite having a comparable number of adult worms [33-35,37,38].

In the present study cohort, co-infected individuals with lower CD4^+^ cell counts levels categorised either at <200 cells/μL or <350 cells/μL had higher GM-epg of *S. mansoni* infection compared to those who had higher CD4^+^ cell count levels. However the difference was not statistically significant. Previous similar studies have reported lower *S. mansoni* egg counts in individuals with lower CD4^+^ cell counts [4,18]. It is important to note that, HIV-1 seropositive individuals included in the present study had higher median CD4^+^ cell counts as compared to similar cohort included in other similar study in rural Zimbabwe, which had very low CD4^+^ cell counts [18]. It is possible that the previous studies, which observed lower *S. mansoni* egg counts in HIV-1 seropositive individuals with lower CD4^+^ cell counts, were focused on cohorts suffering more advanced stages of HIV-1 associated disease [4,18]. It is worthwhile noting that decreased CD4^+^ cell counts have also been reported in HIV-1 seronegative individuals infected with *S. mansoni*, indicating an association between CD4^+^ lymphocytopenia and *S. mansoni* infection [18,39].

In the present study, no direct association or correlation was observed between the geometrical mean *S. mansoni* epg and CD4^+^ cell counts among individuals co-infected with HIV-1 and *S.mansoni*. Our observations were consistent to a similar study in rural Zimbabwe [18]. The results of the present study and those of other authors [18] indicate that even HIV-1 co-infected individuals, regardless of the immune status as measured by CD4^+^ count levels, could have egg counts close to HIV-1 negative individuals infected with *S. mansoni* [18]. In contrast, in co-infected human hosts in western Kenya, lower CD4^+^ cell counts correlated positively with lower egg excretion ratio/CCA ratio [22,32]. The CD4^+^ T-helper cells lymphocyte responses are thought to play a central role in excretion efficiencies of *S. mansoni* eggs, with decreasing CD4^+^ cell counts correlating with reduced excretion efficiencies of *S. mansoni* eggs in murine models [33-38]. It is worth to note that the degree of induced immunonosuppression observed in murine models studies, which resulted in lower *S. mansoni* egg output and correlated positively with CD4^+^ cells, was very severe and cannot be compared to moderately CD4^+^ depleted individuals observed in the present study. Together, the low numbers of co-infected individuals observed in the present study, combined with their relatively modest CD4^+^ depletion, could have reduced the likelihood of observing a significant correlation between CD4^+^ cell count and *S. mansoni* egg output.

Our study was subject to some limitations. The cross-sectional nature of the study design may have contributed to the lack of temporal association between HIV-1, *S. mansoni* and some of the study variables. Thus, even though this study was a community-based study with a large number of study participants, some caution must be taken with the interpretation of these findings.

## Conclusion

In conclusion, no differences in prevalence of *S. mansoni* infection or egg count were observed between HIV-1 sero-positive individuals and HIV-1 sero-negative individuals. In addition, no association was observed between HIV-1 serostatus and prevalence or intensity of infection with *S.mansoni.* Furthermore, no correlation was observed between CD4^+^ cell count levels and *S. mansoni* egg count levels. Our findings suggest that HIV-1 infection may not affect *S. mansoni* disease development and excretion of eggs from the co-infected individuals. Further research is needed to test biological interaction using more sensitive methods of diagnosing *S. mansoni,* such as quantitative CCA, and with a much bigger sample size of co-infected individuals with *S. mansoni* and HIV-1.
